# Strigolactone and abscisic acid synthesis and signaling pathways are enhanced in the wheat oligo-tillering mutant *ot1*

**DOI:** 10.1007/s11032-024-01450-3

**Published:** 2024-02-02

**Authors:** Jiaxing Bai, Huijun Guo, Hongchun Xiong, Yongdun Xie, Jiayu Gu, Linshu Zhao, Shirong Zhao, Yuping Ding, Luxiang Liu

**Affiliations:** State Key Laboratory of Crop Gene Resources and Breeding, National Engineering Laboratory of Crop Molecular Breeding, National Center of Space Mutagenesis for Crop Improvement, Institute of Crop Sciences, Chinese Academy of Agricultural Sciences, Beijing, China

**Keywords:** Wheat, Oligo-tillering, Mutation, Strigolactone, Abscisic acid, Differentially expressed genes

## Abstract

Tiller number greatly contributes to grain yield in wheat. Using ethylmethanesulfonate mutagenesis, we previously discovered the oligo-tillering mutant *ot1*. The tiller number was significantly lower in *ot1* than in the corresponding wild type from the early tillering stage until the heading stage. Compared to the wild type, the thousand-grain weight and grain length were increased by 15.41% and 31.44%, respectively, whereas the plant height and spike length were decreased by 26.13% and 37.25%, respectively. Transcriptomic analysis was conducted at the regreening and jointing stages to identify differential expressed genes (DEGs). Functional enrichment analysis with the Kyoto Encyclopedia of Genes and Genomes (KEGG) and Gene Ontology (GO) databases showed differential expression of genes associated with ADP binding, transmembrane transport, and transcriptional regulation during tiller development. Differences in tiller number in *ot1* led to the upregulation of genes in the strigolactone (SL) and abscisic acid (ABA) pathways. Specifically, the SL biosynthesis genes *DWARF* (*D27*), *D17*, *D10*, and *MORE AXILLARY GROWTH 1* (*MAX1*) were upregulated by 3.37- to 8.23-fold; the SL signal transduction genes *D14* and *D53* were upregulated by 1.81- and 1.32-fold, respectively; the ABA biosynthesis genes *9*-*CIS*-*EPOXICAROTENOID DIOXIGENASE 3* (*NCED3*) and *NCED5* were upregulated by 1.66- and 3.4-fold, respectively; and *SNF1-REGULATED PROTEIN KINASE2* (*SnRK2*) and *PROTEIN PHOSPHATASE 2C* (*PP2C*) genes were upregulated by 1.30- to 4.79-fold. This suggested that the tiller number reduction in *ot1* was due to alterations in plant hormone pathways. Genes known to promote tillering growth were upregulated, whereas those known to inhibit tillering growth were downregulated. For example, *PIN-FORMED 9* (*PIN9*), which promotes tiller development, was upregulated by 8.23-fold in *ot1*; *Ideal Plant Architecture 1* (*IPA1*), which inhibits tiller development, was downregulated by 1.74-fold. There were no significant differences in the expression levels of *TILLER NUMBER 1* (*TN1*) or *TEOSINTE BRANCHED 1* (*TB1*), indicating that the tiller reduction in *ot1* was not controlled by known genes. Our findings provide valuable data for subsequent research into the genetic bases and regulatory mechanisms of wheat tillering.

**Supplementary Information:**

The online version contains supplementary material available at 10.1007/s11032-024-01450-3.

## Introduction

Wheat (*Triticum asetivum* L.) is one of the most important staple crops throughout the world, providing ~ 20% of all food calories consumed by humans (Shiferaw et al. 2013). Wheat yield is determined by the thousand-grain weight, the number of spikes per unit, and the number of grains per spike (Shang et al. 2021). The number of spikes per unit is positively correlated with tiller number. Wheat tillers are lateral branches, that grow from the main stem (Luo et al. 2021). Despite the association between tiller number and yield, lower-tillering mutants can also have high yield. The thousand-grain weights of lines with fewer tillers are increased by an average of 57.43% (Richards 1988), and the yields of oligo-tillering lines are increased by 11.90% compared to multi-tillering lines under drought stress conditions (Mitchell et al. 2013). The *tiller inhibition 4* (*tin4*) mutant exhibits oligo-tillering, but its maximum yield is 2.26–13.33% higher than the yield of multi-tillering lines under high-density planting conditions (Wang et al. 2022). A deeper understanding of the genetic mechanisms underlying wheat tillering could contribute to the genetic improvement of wheat yield.

The genes that control tillering in rice are better understood than those in wheat. *MONOCULM 1* (*MOC1*) and *MONOCULM 3* (*MOC3*) regulate the initiation and outgrowth of axillary buds (Li et al. 2003; Shao et al. 2019). *FLORAL ORGAN NUMBER1* (*FON1*) also regulates axillary bud outgrowth, which promotes tiller growth. *MOC1* and *MOC3* co-activation induces *FON1* expression (Moon et al. 2006; Shao et al. 2019). *LAX PANICLE 1* (*LAX1*) and *LAX PANICLE 2* (*LAX2*) regulate tiller number by promoting differentiation of axillary meristem cells (Oikawa and Kyozuka 2009; Tabuchi et al. 2011). *TEOSINTE BRANCHED 1* (*OsTB1*) encodes the TCP transcription factor, which is primarily expressed in axillary buds. *OsTB1* is a negative regulator of rice lateral branching (Takeda et al. 2003). OsTB1 directly inhibits *OsD14* expression and regulates tiller growth by interacting with the MADS-box domain of MADS57 (Guo et al. 2013). Overexpression of *HEME ACTIVATOR PROTEIN* (*OsHAP2E*) increases the photosynthesis rate and tiller number (Alam et al. 2015). *PALE GREEN LEAF* (*PGL*)/*CAO1* encodes chlorophyllide a oxygenase; *pgl* mutants show inhibited axillary bud growth and decreased tiller number (Yang et al. 2016). *OsbHLH025*/*DPF* encodes a basic helix–loop–helix (bHLH) transcription factor and promotes diterpenoid phytoalexins production. *DPF* overexpression is associated with decreased tiller number (Yamamura et al. 2015). *PIN*-*FORMED* (*OsPIN2*), *OsPIN5b*, and *OsPIN9* regulate auxin transporters; *PIN2* and *PIN9* overexpression increases tiller number (Chen et al. 2012; Hou et al. 2021), whereas *PIN5b* overexpression decreases tiller number (Lu et al. 2015).

Research into wheat tillering has advanced in recent years. Several genes controlling oligo-tillering have been identified in mutants, such as *TILLER NUMBER 1 (tn1)* (Dong et al. 2023), *tin1* (Richards 1988), *tin2* (Peng et al. 1998), *tin3* (Kuraparthy et al. 2006; Ahmed et al. 2023), *tin4* (Wang et al. 2022), *tin5* (Si et al. 2022), *tin6* (Schoen et al. 2023), *fertile tiller inhibition gene* (*ftin*) (Zhang et al. 2013), and *dwarf*-*monoculm* (*dmc*) (An et al. 2019). Morphological studies have revealed that the decreased tiller numbers observed in *tin1*, *tin4*, *tin5*, *ftin*, and *Low number of tillers 1* (*lnt1*) (Dabbert et al. 2010) mutants are due to inhibited and abnormal axillary bud development. In *tin1*, tiller outgrowth ceases when the shoot apex transitions from the vegetative to the reproductive stage (Kebrom et al. 2012; Richards 1988). *ftin* shows a normal tiller number at the seedling stage, but very few tillers are observed at heading stage (Zhang et al. 2013). This suggests that a lower effective tiller number is not due to axillary bud differentiation, but to delayed tiller outgrowth and development at later stages. In *tin4*, *tin5*, and *lnt1*, decreases in tiller number are due to inhibition of secondary tiller bud development (Dabbert et al. 2010; Si et al. 2022; Wang et al. 2022). In contrast, both tiller bud differentiation and development are inhibited in *dmc* (An et al. 2019). In *tin6*, tiller number is decreased at the seedling and heading stages (Schoen et al. 2023). Genes that regulate wheat tillers have pleiotropic effects for plant height and other yield related traits. For example, significantly higher thousand-grain weight, grain length, and grain width have been identified in lower-tillering wheat mutants (Kuraparthy et al. 2006; Wang et al. 2022).

Strigolactones (SLs) are phytohormones produced from plant carotenoid derivatives (Al-Babili and Bouwmeester 2015). SLs and their precursors affect many aspects of plant architecture and development, including tiller formation and development and shoot branching. A lack of SLs can increase tiller and shoot branching numbers, whereas excessive SL levels can inhibit tiller growth (Gomez-Roldan et al. 2008; Zhao et al. 2019). Excessive SL levels also promote lateral root formation and hair elongation (Kapulnik et al. 2011; Sun et al. 2016). Furthermore, SLs play important roles in rhizosphere signaling through effects such as inducing hyphal branching among arbuscular mycorrhizal fungi and stimulating germination of the parasitic plants *Striga* spp. and *Orobanche* spp. (Fiorilli et al. 2019; Zhao et al. 2019). Several genes in SL biosynthesis and signal transduction pathways have been identified in rice and *Arabidopsis thaliana*. SL biosynthesis is regulated by four main enzymes. The first step is reversible isomerization of all-trans-into 9-cis-β-carotene, which is catalyzed by DWARF27 (D27) (Abuauf et al. 2018). The product undergoes cleavage and rearrangement by CAROTENOID CLEAVAGE DIOXYGENASE 7 (CCD7)/D17 and CAROTENOID CLEAVAGE DIOXYGENASE 8 (CCD8)/D10, respectively, yielding carlactone (Alder et al. 2012). Carlactone is catalyzed of cytochrome P450 monooxygenase, after which MORE AXILLARY GROWTH 1 (MAX1) and other enzymes act sequentially to form canonical and non-canonical SLs such as carlactonoic acid, 4-deoxyorobanchol, and Orobanchol (Booker et al. 2005). D3, D14, and D53 are involved in SL signal transduction (Ishikawa et al. 2005; Jiang et al. 2013; Zhao et al. 2013), which is initiated by the α/β-sheet hydrolase receptor portion of protein D14. D14 binds D3 through the C-terminal helix structure (Shabek et al. 2018), then combines with D53 to form a complex for SL signal transduction (Tal et al. 2022; Zhou et al. 2013). *CYTOKININ OXIDASE/DEHYDROGENASE 9* (*CKX9*) and *Ideal Plant Architecture 1* (*IPA1*) are the downstream genes that directly participate in SL signal transduction (Duan et al. 2019; Song et al. 2017).

Abscisic acid (ABA) is derived from β-carotene through a series of enzymatic reactions. This process begins with hydroxylation of all-trans-β-carotene and results in zeaxanthin formation. Subsequently, ABA DEFICIENT 1 (ABA1) converts all-trans-zeaxanthin to all-trans-violaxanthin (Barrero et al. 2005). ABA DEFICIENT 4 (ABA4) is involved in the conversion of all-trans-violaxanthin to all-trans-neoxanthin (Dall’Osto et al. 2007), and an ABA4 homolog affects branching and adventitious root formation (Ma et al. 2014). Carotenoids with the all-trans configuration are then isomerized to the 9-cis counterparts, which are catalyzed by 9-CIS-EPOXICAROTENOID DIOXIGENASE 3 (NCED3) and 9-CIS-EPOXICAROTENOID DIOXIGENASE 5 (NCED5) to form xanthoxin (Bang et al. 2013; Zhu et al. 2009). Xanthoxin is further oxidized by ABA DEFICIENT 2 (ABA2) to produce abscisic aldehyde; this is in turn oxidized by abscisic aldehyde oxidases (AAOs) and ABA DEFICIENT 3 (ABA3) to produce ABA (Nambara et al. 1998; Watanabe et al. 2018). PYR-LIKE (PYLs) and PROTEIN PHOSPHATASE 2C (PP2Cs) also play crucial roles in ABA perception and signal transduction (Bhatnagar et al. 2017; Kim et al. 2012). ABA forms complexes with PYLs and PP2Cs, enabling the release of PP2C-mediated SNF1-REGULATED PROTEIN KINASE 2 (SnRK2) inhibition. PYLs, PP2Cs, and SnRK2s are the three core protein elements in the ABA signaling pathway (Chen et al. 2020).

RNA-sequencing (RNA-seq) has been widely used to identify differentially expressed genes (DEGs) between biological samples. DEGs can reveal the genetic mechanisms underlying phenotypic changes. In the present study, an oligo-tillering mutant, *ot1*, was developed in the winter wheat cultivar “Jing411” via ethyl methane sulfonate (EMS) treatment. Compared with the corresponding wild type (WT), *ot1* exhibited decreased tiller number and plant height but increased thousand-grain weight. The tiller bud tissues of *ot1* and WT plants were sampled and characterized at the transcriptomic level via RNA-seq. This analysis suggested that the tiller number inhibition in *ot1* may have been correlated with the SL and ABA pathways. The results of this study could guide future research into the regulatory mechanisms controlling wheat tillering.

## Materials and methods

### Plant materials

The oligo-tillering mutant *ot1* was discovered in a previously generated EMS population (Guo et al. 2017) using the winter wheat cultivar “Jing411” as the parent. *ot1* was purified through multiple generations of self-pollination; the stable M_7_ line was used in transcriptomic and pathway regulation analyses. This mutant was also crossed with the WT, to develop the multi-tiller and oligo-tiller BC_2_F_2_ and BC_2_F_3_ lines, which were used to verify gene expression patterns. The mutant, WT, and BC_2_F_3_ lines were planted at the experimental station of the Institute of Crop Sciences, Chinese Academy of Agricultural Sciences (Beijing, China). Ten rows of the mutant and WT were planted and two rows of each BC_2_F_3_ line were planted. Each row was 2 m length and there was 10 cm between individual plants.

### Investigation of tillering and other agronomic traits

Tiller number was counted in 10 *ot1* and 10 WT plants from the regreening stage until the heading stage. After harvesting, 10 individuals of each genotype were selected for measurements of plant height, spike length, thousand-grain weight, grain width, and grain length. Tiller number was counted in the BC_2_F_3_ lines at 66 d after sowing for 10 individuals from each line. These values were compared with those of the corresponding parents.

### Statistical analyses

All statistical analyses were conducted in GraphPad Prism 8 v8.0.2. Differences between pairs of groups were assessed with Student’s *t* test and considered significant at *p* < 0.05.

### Sampling for RNA-seq

Axillary buds were collected from *ot1* and WT plants at the regreening stage (141 d after sowing) and the jointing stage (170 d after sowing). The tiller buds of four individual plants were collected and mixed to form a single sample; there were three replicates per genotype at each stage for a total of 12 samples. These were used for RNA-seq and validation with real-time quantitative PCR (RT-qPCR). RNA was extracted using the RNeasy Plant Mini Kit (Qiagen, Germany). RNA quantity and integrity were measured with a Nanodrop 2000 (Thermo Fisher Scientific, Waltham, MA, USA) and a Bioanalyzer 2100 (Agilent Technologies, Santa Clara, CA, USA), respectively.

The tiller buds of multi-tiller and oligo-tiller BC_2_F_3_ lines and their parents were also collected at the seedling stage (66 d after sowing) for RT-qPCR verification. For this, three BC_2_F_3_ lines were combined to form a single sample.

### RNA-seq and related analyses

RNA-seq libraries were constructed using the Illumina RNA Library Prep Kit (Vazyme, Nanjing, China) following the manufacturer’s instructions. Poly(A)-containing transcripts were enriched from the total RNA with Oligo(dT)-coated magnetic beads. For each sample, adapters with unique barcodes were ligated to the end-polished cDNA fragments. The libraries were amplified with PCR and quantitated on a Qubit 2.0 (Thermo Fisher Scientific, Waltham, MA, USA). RNA libraries were sequenced on the Illumina NovaSeq platform, producing raw reads in FASTQ format. Sequencing was conducted by Tcuni (Chengdu, China). Adapters and low-quality data were removed using fastp (https://github.com/OpenGene/fastp) with default parameters. The Q30 scores and GC contents of the clean data were calculated simultaneously. Principal component analysis (PCA) was conducted on the clean data using BWKCloud tools (https://www.biocloud.net/fxpt/app).

Quantitative sample analysis was performed with kallisto (https://pachterlab.github.io/kallisto/) using the default parameters (Bray et al. 2016) to obtain gene expression values. Differences between sample types were then analyzed with the R package “edgeR3” (https://bioconductor.org/packages/release/bioc/html/edgeR.html) using thresholds of false discovery rate (FDR) ≤ 0.05 and |Log_2_(fold change [FC])| ≥ 1 (Robinson et al. 2010). The resulting DEGs were used in subsequent functional analyses.

Gene Ontology (GO) term enrichment analyses were conducted with the R package “Goseq” (Young et al. 2010), and Kyoto Encyclopedia of Genes and Genomes (KEGG) biochemical pathway enrichment analyses were carried out with KOBAS (Xie et al. 2011). GO and KEGG terms enriched at a corrected *p* value < 0.05 were considered statistically significant.

### RT-qPCR

Samples used in RNA-seq were validated with RT-qPCR. After genomic DNA was removed from the RNA samples, first-strand cDNA was synthesized using the All-in-One First-Strand cDNA Synthesis SuperMix Kit (TransGen Biotech, China). RT-qPCR was then conducted with the PerfectStart Green qPCR SuperMix Kit (TransGen Biotech, China) on the CFX 96 Real-Time System (Bio Rad, Hercules, CA, USA) with following thermocycling program: one cycle of 30 s at 94 ℃ followed by 45 cycles of 5 s at 94 ℃, 15 s at 60 ℃, and 10 s at 72 ℃. A melting curve analysis was conducted at the end of the 45 cycles. Reverse transcription and qPCR procedures were performed following the manufacturers’ instructions. RT-qPCR was carried out in technical triplicate. Relative expression levels were calculated using the 2^−ΔΔCt^ method (Livak and Schmittgen 2001) with normalization to the internal control gene *TaActin*. All primers used for RT-qPCR are shown in Table S1.

### Expression correlation coefficient analysis

Correlations of expression levels of DEGs between the RNA-seq and RT-qPCR data were calculated. The fold change of DEGs in the mutant and WT from RNA-seq or RT-qPCR was calculated by gene expression levels in the mutant divided by that in the WT. The correlations of RNA-seq and RT-qPCR data were analyzed using GraphPad Prism 8 v8.0.2. Correlations of gene expression between BC_2_F_3_ lines and the corresponding parent lines from the RT-qPCR data were also calculated based on the gene expression levels from oligo-tiller lines divided by that from multi-tiller lines.

## Results

### *ot1* had a significantly lower-tiller number but increased thousand-grain weight compared to the WT

**Fig. 1:**
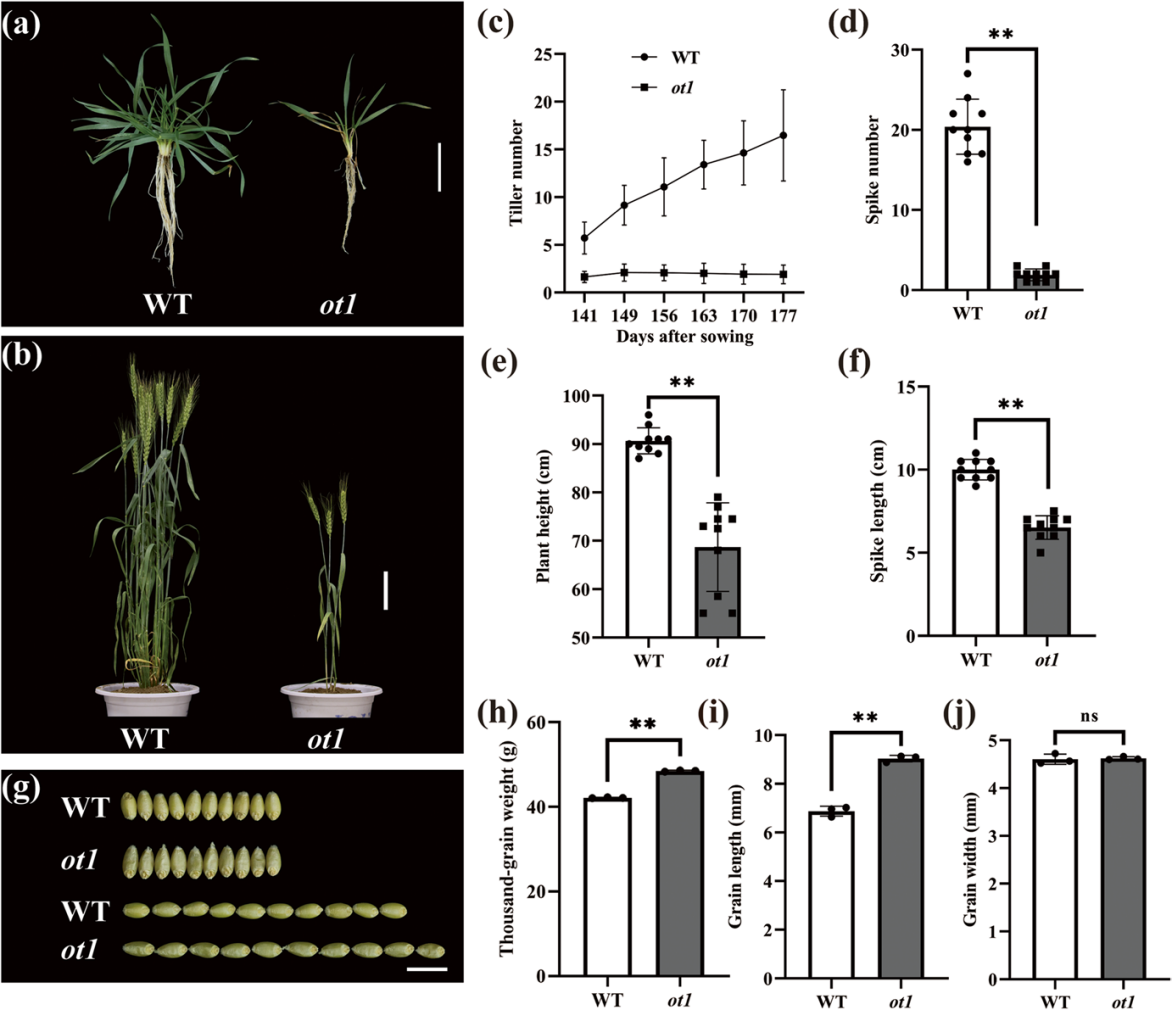
Phenotypic variations in *ot1*. **a** *ot1* and wild-type (WT) at the seedling stage. Scale bar = 5 cm. **b** *ot1* and WT plants at the heading stage. Scale bar = 20 cm. **c** Dynamic changes in tiller number from the regreening stage to the heading stage. **d** Effective tiller number at the heading stage. **e** Plant height. **f** Spike length. **g** Grain length and width. Scale bar = 1 cm. **h** Thousand-grain weight. **i** Grain length. **j** Grain width. Data are presented as the mean ± standard deviation (SD). *p < 0.05, **p < 0.01; ns, no significant difference (two-tailed Student’s *t*-test)

The mutant line *ot1* exhibited fewer tillers than the corresponding WT (Fig. 1a, b). From the seedling until the heading stage, the tiller number did not significantly increase in *ot1* as the plants grew (Fig. 1c). Furthermore, the *ot1* spike number was only 9.31% of the spike number in the WT at the heading stage (Fig. 1d).

*ot1* also showed a significantly decreased plant height and spike length compared to the WT (Fig. 1e, f). Specifically, the average plant height was 66.86 cm, representing a 26.13% decrease compared to the WT, and the *ot1* spike length was decreased by 37.25%. However, the thousand-grain weight and grain length were increased by 15.41% and 31.44%, respectively, compared to the WT (Fig. 1g–i). No significant variation was detected in grain width (Fig. 1j). This indicated that grain length but not width contributed to the increased thousand-grain weight of *ot1* mutants.

### RNA-seq data were of high quality

A total of 162.60 Gb of clean data were obtained, ranging from 12.56 to 15.05 Gb per sample. The GC content of the 12 samples ranged from 52.50 to 53.20% and the average Q30 coverage was 92.33% (Table S2). These results indicated that the RNA-Seq data were suitable for further analyses.

### DEGs between *ot1* and WT

At the regreening stage, 5582 DEGs were identified in *ot1* compared to WT plants. Of these, 4865 were upregulated and 717 were downregulated (Table S3). *TraesCS6B03G1086300LC* was the most strongly downregulated gene in the *ot1* with a Log_2_(FC) value of 8.74. This gene encodes a protein of unknown function. The most strongly upregulated gene in *ot1* was *TraesCSU03G0251900*, which encodes a protein modifier of SNC11-like.

**Fig. 2:**
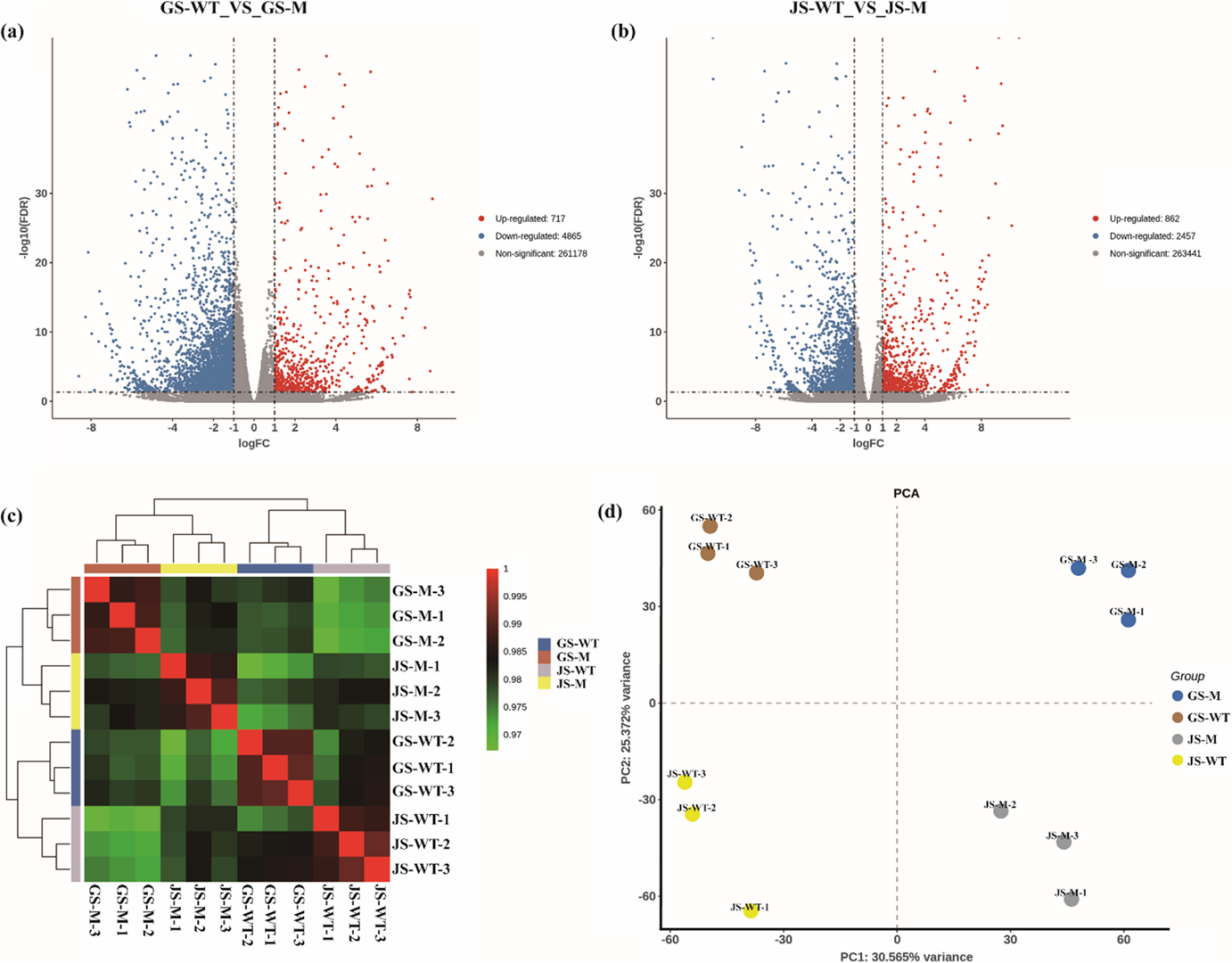
Transcriptomic analysis at the regreening and jointing stages. **a** Relative expression of differentially expressed genes (DEGs) at the regreening stage. **b** Relative expression of DEGs at the jointing stage. **c** Correlation analysis of expression levels between *ot1* and WT plants. **d** Principal component analysis (PCA) of *ot1* and WT plants at each sampled stage. GS, regreening stage; JS, jointing stage

At the jointing stage, there were 3319 DEGs in *ot1* compared to WT plants (Fig. 2a, b). Among them, 2457 were upregulated and 862 were downregulated (Table S4). *TraesCS6A03G1022000LC* was the most strongly downregulated in *ot1*; this gene encodes a protein of unknown function. *TraesCS2B03G0186800LC*, which also encodes a protein of unknown function, was the most strongly upregulated gene in *ot1*.

Cluster analysis indicated that the gene expression patterns among biological replicates were similar (Fig. 2c), demonstrating the replicability and reliability of the transcriptomic data. PCA also showed significant differences between the four groups. The first and second principal components (PC1 and PC2, respectively) accounted for 30.56% and 25.37% of the variance, respectively (Fig. 2d).

### Genes involved in ADP binding, transmembrane transport, and transcriptional regulation were differentially expressed in *ot1*

To further explore biological pathways or processes related to wheat tillering, GO enrichment analysis was performed on the DEGs at both stages. GO terms were assessed in three categories: biological process (BP), molecular function (MF), and cellular component (CC) terms.

**Fig. 3:**
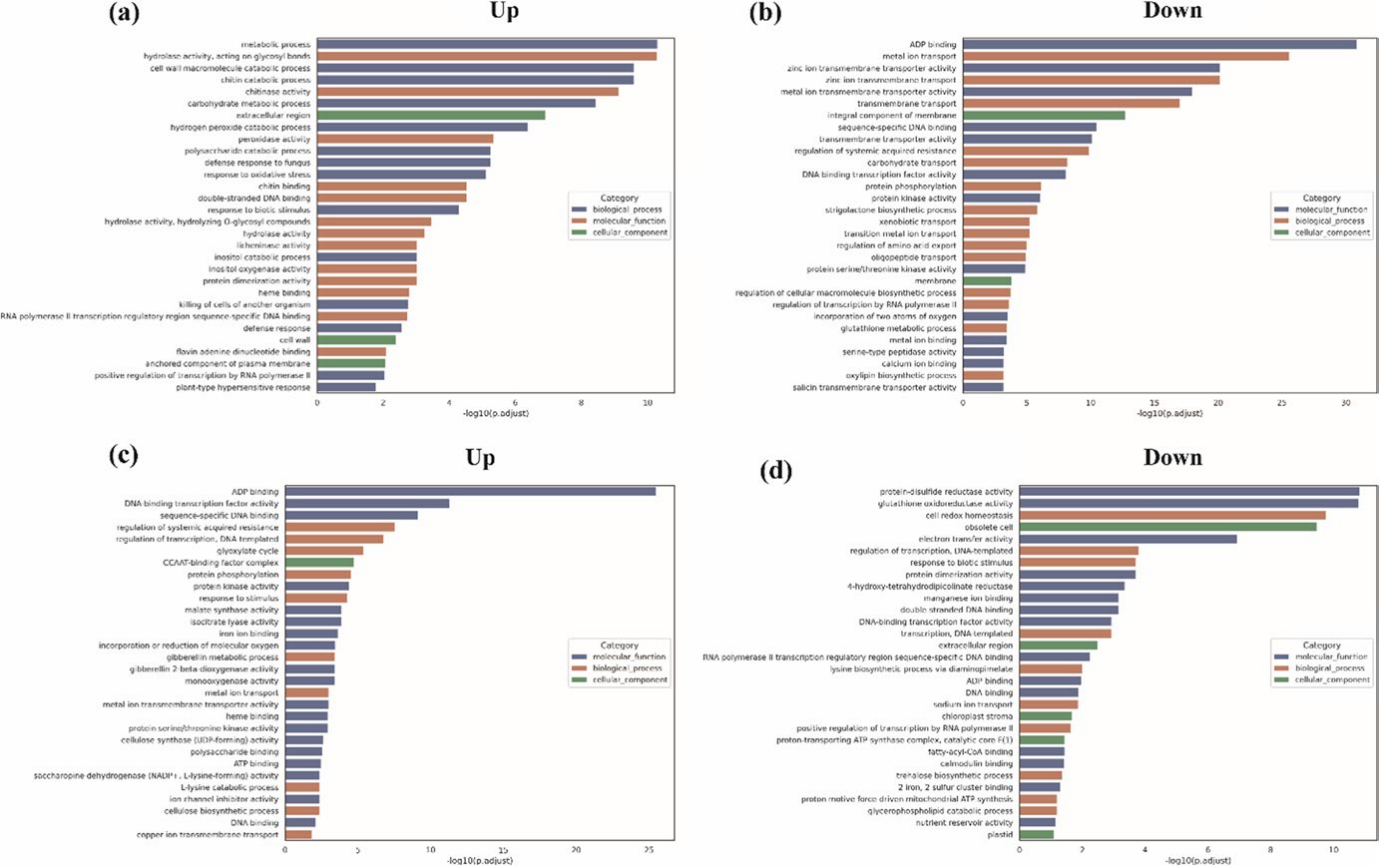
Significantly enriched Gene Ontology (GO) terms in DEGs between *ot1* and WT plants at the regreening and jointing stages. **a**, **b** Significantly enriched biological process (BP), molecular function (MF), and cellular component (CC) terms in the **a** upregulated and **b** downregulated DEGs at the regreening stage. **c**, **d** Significantly enriched BP, MF, and CC terms in the **c** upregulated and **d** downregulated DEGs at the jointing stage. GS, regreening stage; JS, jointing stage

At the regreening stage, the upregulated DEGs were classified into 377 BP terms, 354 MF terms, and 72 CC terms (Table S5). The most significantly enriched were the BP terms, which included metal ion transport, zinc ion transmembrane transport, and transmembrane transport (Fig. 3a). Among the downregulated DEGs, 121 BP terms, 141 MF terms, and 44 CC terms were significantly enriched (Table S6). Here again, the BP terms were most significantly enriched and included the terms “metabolic process,” “cell wall macromolecule catabolic process,” and “chitin catabolic process.” Genes related to ADP binding and transmembrane transport were also significantly enriched, as were genes associated with ion transmembrane transport, the cell membrane, and cell wall composition (Fig. 3b).

At the jointing stage, the upregulated DEGs were significantly enriched in BP terms, including regulation of systemic acquired resistance, transcription, and the glyoxylate cycle (Fig. 3c). The most highly enriched BP terms in the downregulated DEGs were cell redox homeostasis, regulation of transcription, and response to biotic stimulus (Fig. 3d). Genes associated with ADP binding, transcription regulation, and DNA binding were significantly upregulated in the axillary buds. This suggested that tiller bud growth was regulated by various transcription factors at this stage. *HAP2E* and *MOC1* are transcription factors associated with transcriptional regulation and DNA binding; these genes are known to control tiller growth (Li et al. 2003; Alam et al. 2015). Here, the homeologs *HAP2E*-*A* and *HAP2E*-*B* were upregulated in *ot1* mutants by 1.52-fold and 1.58-fold, respectively, and *MOC1*-*D* was upregulated by 1.12-fold compared to the WT.

### *ot1* mutation altered metabolic and phytohormone signal transduction pathways

**Fig. 4:**
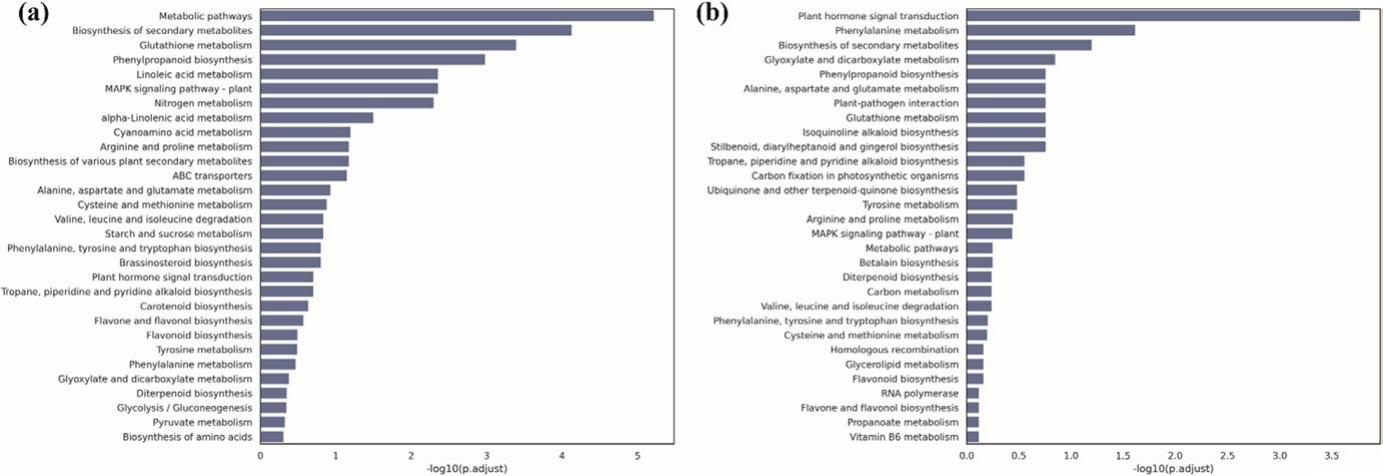
Significantly enriched Kyoto Encyclopedia of Genes and Genomes (KEGG) biochemical pathways among DEGs between *ot1* and WT plants at the regreening stage and jointing stages. **a** Enriched KEGG pathways among DEGs at the regreening stage. **b** Enriched KEGG pathways among DEGs at the regreening stage. GS, regreening stage; JS, jointing stage

KEGG biochemical pathway enrichment analyses were next performed in the DEGs at both stages to explore biochemical pathways involved in tiller growth. At the regreening stage, the 5582 DEGs were involved in 117 pathways (Table S7). The 10 most highly enriched pathways were metabolic pathways (taes01100), biosynthesis of secondary metabolites (taes01110), glutathione metabolism (taes00480), phenylpropanoid biosynthesis (taes00940), linoleic acid metabolism (taes00591), the MAPK signaling pathway (taes04016), nitrogen metabolism (taes00910), alpha-Linolenic acid metabolism (taes00592), cyanoamino acid metabolism (taes00460), and arginine and proline metabolism (taes00330) (Fig. 4a). At the jointing stage, the 3319 DEGs were annotated as involved in 109 pathways (Table S8). The 10 most strongly enriched pathways were plant hormone signal transduction (taes04075), phenylalanine metabolism (taes00360), biosynthesis of secondary metabolites (taes01110), glyoxylate and dicarboxylate metabolism (taes00630), phenylpropanoid biosynthesis, alanine (taes00940), aspartate and glutamate metabolism (taes00250), plant-pathogen interaction (taes04626), glutathione metabolism (taes04626), isoquinoline alkaloid biosynthesis (taes00480), and stilbenoid, diarylheptanoid, and gingerol biosynthesis (taes00950) (Fig. 4b).

### Differential expression of genes related to phytohormone synthesis and signal transduction in *ot1*

DEGs related to the SL, ABA, indole-3-acetic acid (IAA), ethylene (ETH), and jasmonate (JA) biosynthesis and signal transduction pathways were identified. The expression levels of selected DEGs were validated by RT-qPCR. Correlation analysis of expression level of each gene from RNA-seq and RT-qPCR data revealed that a high correlation (*R*^2^ = 0.85) was observed between these two data (Fig S1a, b).

**Fig. 5:**
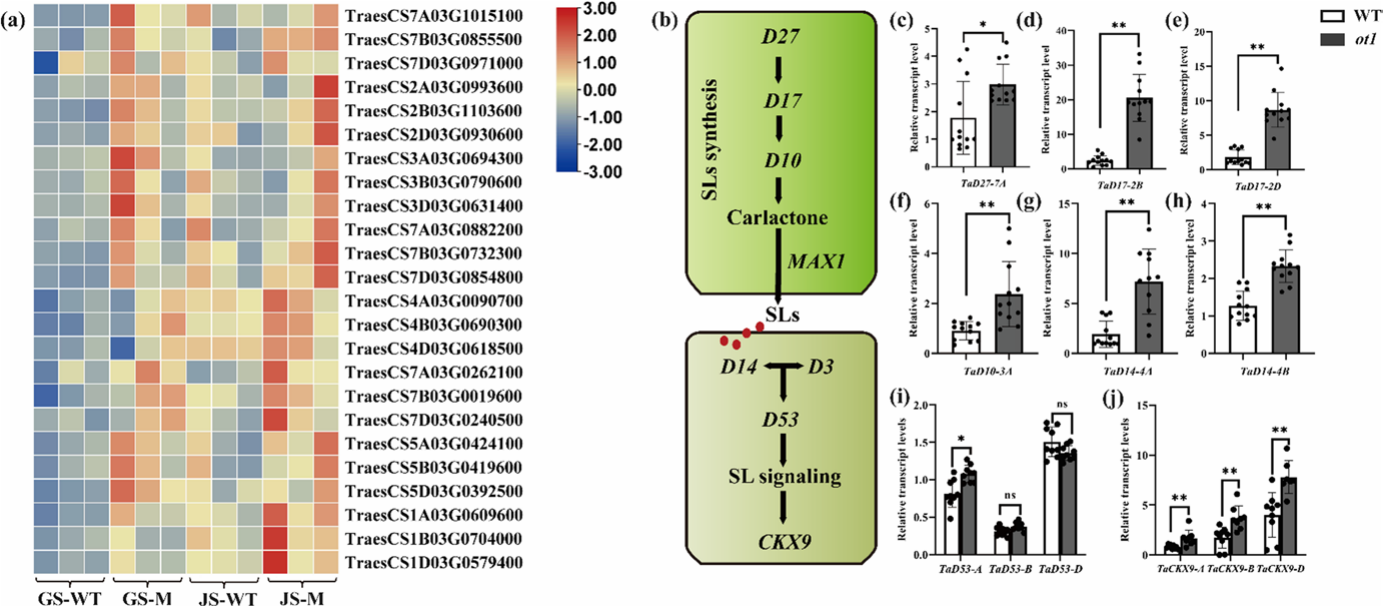
Expression levels of genes related to strigolactone (SL) biosynthesis and signal transduction as determined with real-time quantitative PCR (RT-qPCR). **a** Expression levels of genes involved in SL pathways at the regreening and jointing stages. **b** Model of the SL biosynthesis and signaling pathways. **c**–**j** Expression levels of **c** *TaD27-7A*, **d** *TaD17-2B*, **e** *TaD17-2B*, **f** *TaD10-3A*, **g** *TaD14-4A*, **h** *TaD14-4B*, **i** *TaD53-A*, *TaD53-B*, and *TaD53-D*, and **j** *TaCKX9-A*, *TaCKX9-B*, and *TaCKX9-D*. GS, regreening stage; JS, jointing stage. Color indicates expression values in Log_2_(TPM). Data are presented as the mean ± SD, *p < 0.05, **p < 0.01 (two-tailed Student’s *t*-test)

Transcriptomic analysis revealed significant upregulation of the carotenoid oxygenase gene in *ot1* (Table S9). Because both SL and ABA are generated from carotenoid derivatives (Barrero et al. 2005; Yoneyama et al. 2018), we further investigated the expression levels of genes involved in the ABA and SL biosynthesis and signaling pathways. For these pathways, expression levels of some genes were also validated with RT-qPCR (Fig. 5, Table S10). Compared with the WT, the SL biosynthesis genes *TaD27-A*, *TaD27-B*, and *TaD27-D* were significantly upregulated (by 1.96- to 3.37-fold) in *ot1*; *TaD17-A*, *TaD17-B*, and *TaD17-D* were upregulated by 1.79- to 4.27-fold, and *TaD10-A*, *TaD10-B*, and *TaD10-D* were upregulated by 5.71-, 2.67-, and 5.34-fold, respectively (Fig. 5c–f). Furthermore, *TaMAX1-A*, *TaMAX1-B*, and *TaMAX1-D* were significantly upregulated by 3.94- to 8.23-fold in *ot1*. This suggested that genes involved in SL biosynthesis were expressed at significantly higher levels in *ot1* than WT plants. The SL signal transduction *TaD14* and *TaD53* genes were also significantly upregulated. Specifically, *TaD14-A*, *TaD14-B*, *TaD14-D*, and *TaD53-A* were significantly upregulated by 1.44-, 1.81-, 1.23-, and 1.32-fold, respectively (Fig. 5g–i). *CKX9* is a downstream gene of D53; the wheat subgenomes A, B, and D contain three homeologs of *CKX9* namely *TaCKX9-A*, *TaCKX9-B*, and *TaCKX9-D*, respectively. These three genes were significantly upregulated by 2.07-, 2.16-, and 1.95-fold, respectively (Fig. 5j).

These results suggested that the expression levels of genes associated with the SL pathway were significantly altered in *ot1* mutants, which had abnormal tiller development. Furthermore, compared with the regreening stage, *TaMAX1*, *TaD14*, and *TaCKX9* were upregulated by 37.27%, 37.40%, and 122.23%, respectively at the jointing stage.

**Fig. 6:**
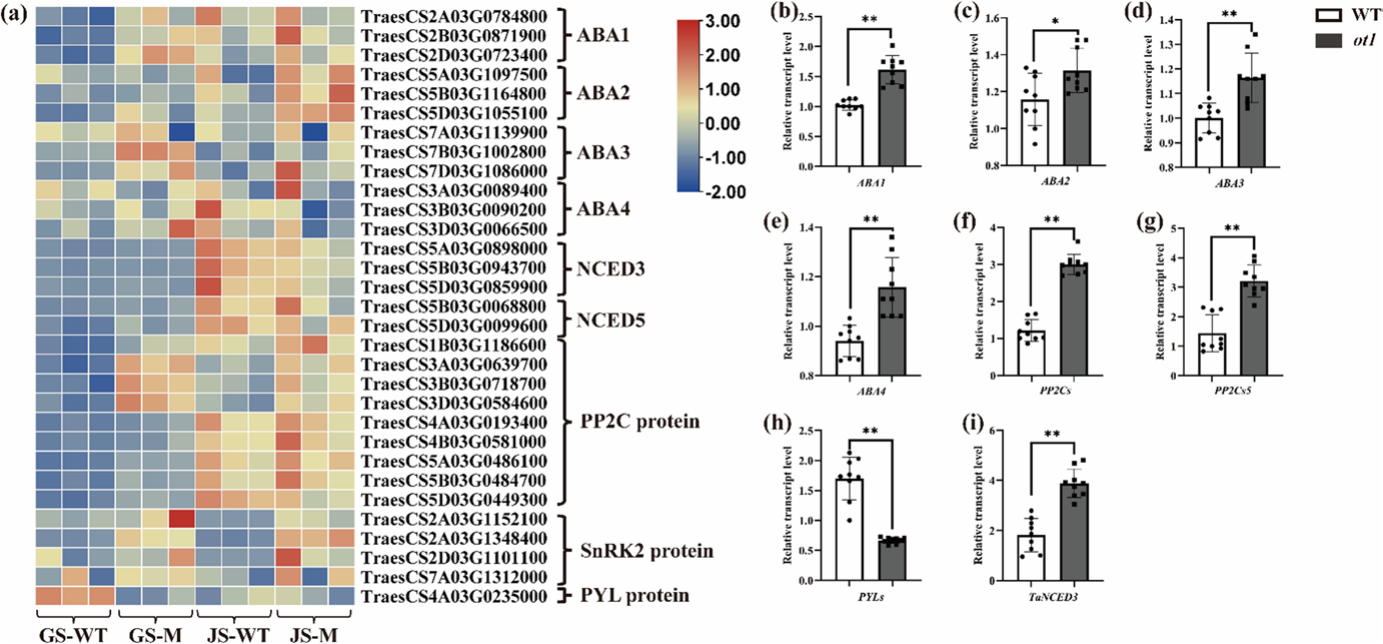
Expression levels of DEGs associated with abscisic acid (ABA) biosynthesis and signal transduction and as determined with RT-qPCR. **a** Expression levels of genes involved in the ABA pathway at the regreening and jointing stages. **b**–**i** Expression levels of **b** *ABA1*, **c** *ABA2*, **d** *ABA3*, **e** *ABA4*, **f** *PP2C*s, **g** *PP2C*s*5*, **h** *PYL*, and **i** *TaNCED3*. GS, regreening stage; JS, jointing stage. Color indicates expression values in Log_2_(TPM). Data are presented as the mean ± SD. *p < 0.05, **p < 0.01 (two-tailed Student’s *t*-test)

It has previously been demonstrated that ABA can inhibit the growth and development of lateral buds. The ABA biosynthesis genes *ABA1*, *ABA2*, *ABA3*, and *ABA4* were significantly upregulated in *ot1* compared to the WT (Fig. 6b–e). *NCED3* and *NCED5* are reportedly involved in the ABA biosynthesis pathway (Dong et al. 2023; Zhu et al. 2009); these two genes were upregulated by 1.66- and 3.4-fold, respectively, in *ot1* compared to the WT (Fig. 6a, i). Furthermore, in *ot1*, the average *NCED3* and *NCED5* expression levels at the jointing stage were 4.81- and 3.23-fold higher, respectively, than in the regreening stage. Genes involved in ABA signal transduction, such as *PP2C*s and *SnRK2*s, were also significantly upregulated in *ot1* compared to the WT (Fig. 6f, g). For example, *PP2C*s were upregulated by 1.9- to 4.79-fold, and *SnRK2*s were upregulated by 2.09- to 2.79-fold. In contrast, *PYL* was significantly downregulated by 70% (Fig. 6h, Table S11). These differences in gene expression between genotypes suggested that the ABA biosynthesis and signaling pathways may have been enhanced in *ot1*. There were no significant differences in *PYL*, *PP2C*, or *SnRK2* expression levels in *ot1* between the regreening stage and the jointing stage.

There was a total of 111 DEGs related to auxin biosynthesis and signaling in *ot1* (Table S12). These included 13 IAA biosynthesis genes; of seven such genes encoding flavin monooxygenase-like proteins, four were upregulated and three were downregulated. The other six genes, which encoded aldehyde oxidase/xanthine dehydrogenase proteins, were all upregulated. The remaining 98 genes were involved in auxin signaling and comprised 11 *PIN*s involved in IAA transport, 21 genes encoding AUX/IAA proteins as auxin response factors, 51 genes in the auxin-responsive *SAUR* gene family, nine genes encoding GH3 family proteins, and six genes encoding auxin-repressed proteins.

DEGs in the ETH and JA pathways were also investigated. Two genes encoding ethylene-responsive binding factors and four genes encoding ethylene insensitive protein were significantly upregulated in *ot1* (Table S13). Additionally, 36 genes encoding proteins with the Tify domain that were involved in the JA pathway were significantly upregulated in *ot1* (Table S14). Such differences in the expression levels of genes associated with hormone synthesis and signaling pathways could partially explain the oligo-tillering phenotype of *ot1*.

### Expression levels of known tillering development regulatory genes were not consistent with tiller variations

**Fig. 7:**
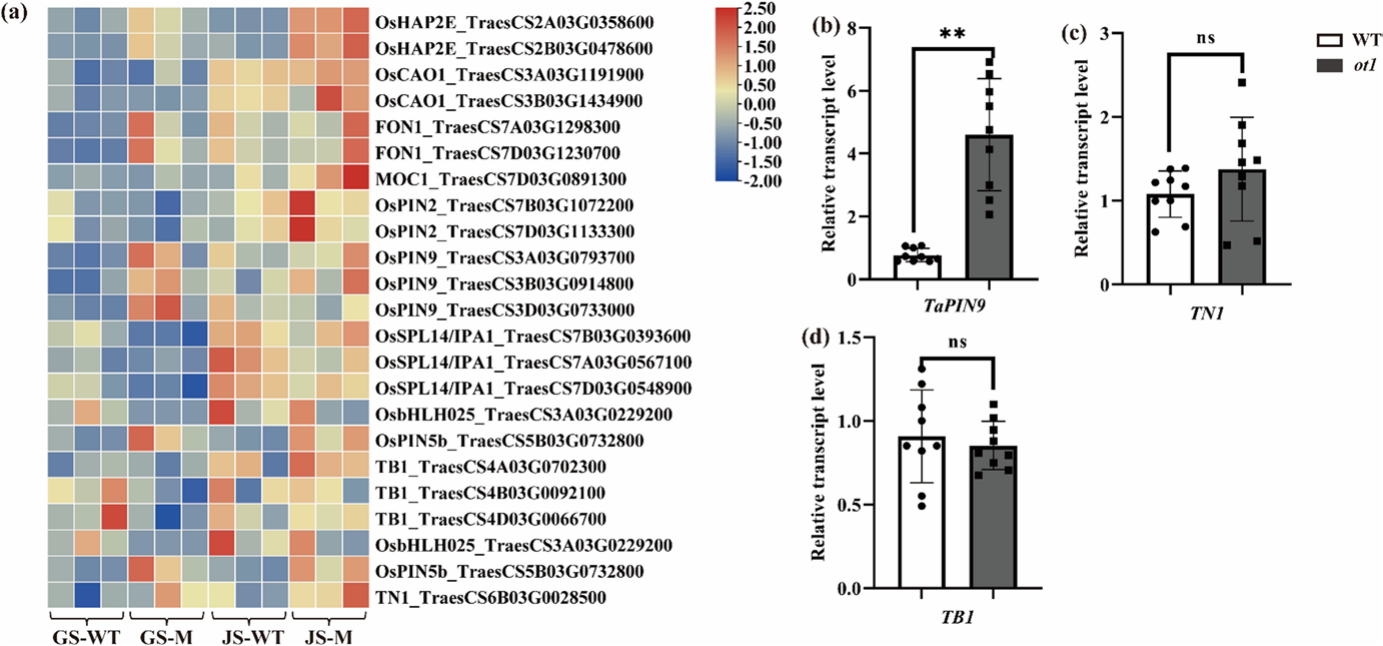
Expression levels of DEGs involved in tiller development as determined with RT-qPCR. **a** Expression levels of genes involved in the ABA pathway at the regreening and jointing stages. **b**–**d** Expression levels of **b** *TaPIN9*, **c** *TN1*, and **d** *TB1*. GS, regreening stage; JS, jointing stage. Color indicates expression values in Log_2_(TPM). Data are presented as the mean ± SD. *p < 0.05, **p < 0.01 (two-tailed Student’s *t*-test)

We also examined the expression levels of other genes known to be involved in tiller development (Table S15, Fig. 7a–d). Intriguingly, compared with WT, expression levels of genes known to promote tiller development were significantly upregulated in the mutant, whereas genes known to inhibit tiller development were significantly downregulated. For example, *HAP2E*, *PIN2*, *PIN9*, *CAO1*, *MOC1*, and *FON1*, which are known to promote tillering in rice, were all significantly upregulated (from 1.11- to 8.23-fold) in *ot1* (Fig. 7a, b). The genes *IPA1*, *PIN5b*, and *bHLH025* are known to inhibit tillering in rice; here, *IPA1* and *bHLH025* were significantly downregulated in *ot1*, although *PIN5b* was significantly upregulated (by 3.70-fold). There were no significant differences in *TN1* or *TB1* expression (Fig. 7c, d).

Although there were no significant differences in the expression of *TB1* between *ot1* and WT plants at either stage, expression of this gene did gradually increase along with tiller development. Compared to the regreening stage, *TB1* was upregulated by 1.52-fold at the jointing stage. Similarly, *PIN2*, *IPA1*, and *MOC1* were upregulated by an average of 2.92-, 2.18-, and 3.16-fold, respectively, at the jointing stage.

### Gene expression patterns in BC_2_F_3_ lines were consistent with those of the corresponding parents

**Fig. 8:**
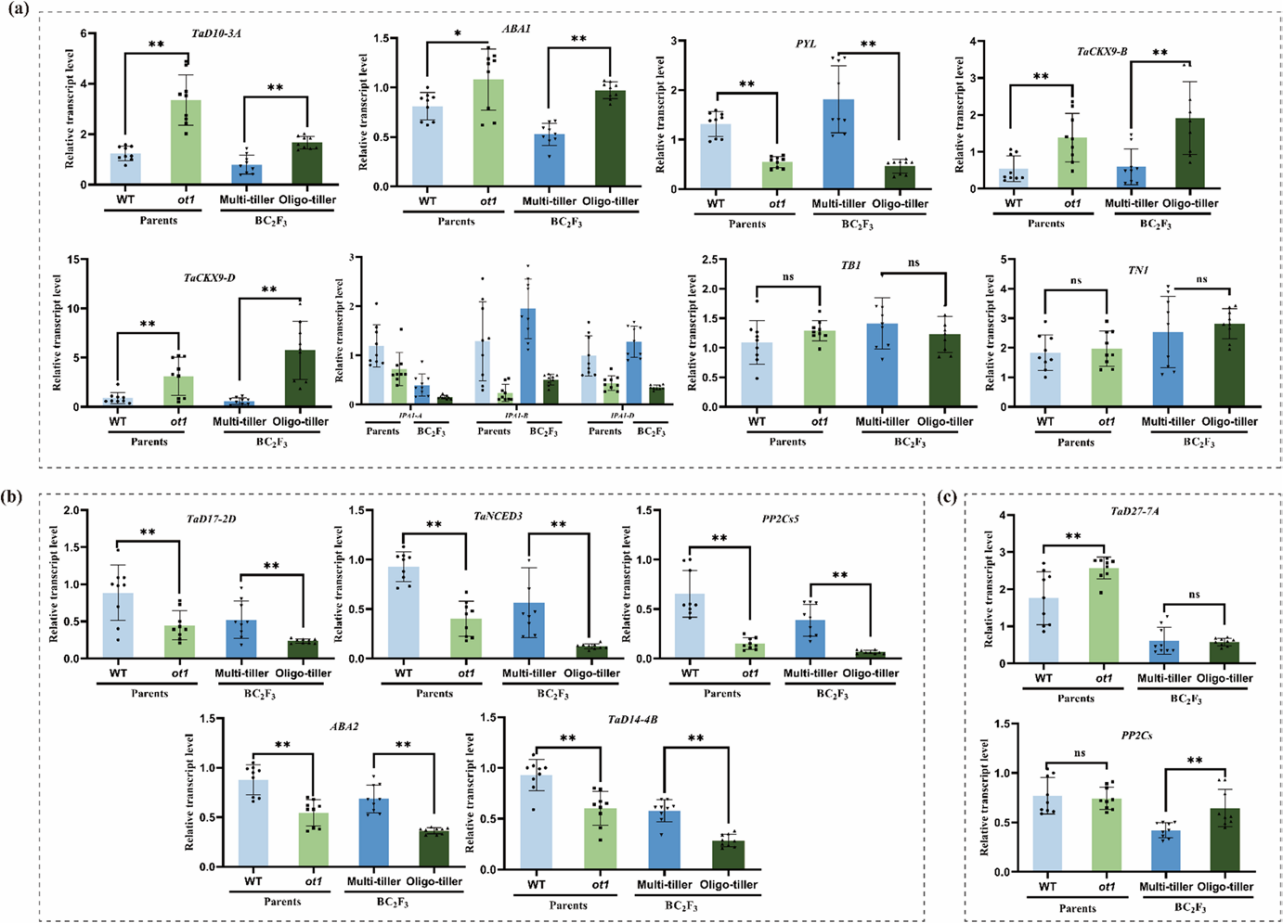
Expression patterns in BC_2_F_3_ lines as determined with RT-qPCR. **a** Genes with consistent expression patterns between multi-tiller and oligo-tiller lines and across sampling stages. **b** Genes with consistent expression patterns between multi-tiller and oligo-tiller lines. **c** Genes with inconsistent expression patterns. Data are presented as the mean ± SD. *p < 0.05, **p < 0.01; ns, no significant difference (two-tailed Student’s *t*-test)

The BC_2_F_3_ populations with oligo- and multi-tillers (Fig S2) were further used to validate the relationship between gene expression levels and tillering. Correlation analysis showed that expression levels of key genes were highly correlated between the BC_2_F_3_ lines and the corresponding parents (Fig S1c, d). There were no significant differences detected in *TN1* or *TB1* between the multi- and oligo-tiller lines. Expression levels of eight genes (*TaD10-3A*, *ABA1*, *PYL*, *TaCKX9-B*, *TaCKX9-D*, *IPA1-A*, *IPA1-B*, and *IPA1-D*) were consistent between the BC_2_F_3_ lines and their parents (Fig. 8a). Expression levels of five genes (*TaD17-2D*, *TaD14-4B*, *ABA2*, *TaNCED3*, and *PP2C*s*5*) were not consistent with the transcriptomic data, but were consistent with the corresponding parents (Fig. 8b). This suggested that those genes may also have been regulated by the growth period. The expression patterns of *TaD27-7A* and *PP2C*s were inconsistent between the two sampling stages and among genotypes (Fig. 8c), suggesting that they may not have regulated tiller development in *ot1*.

## Discussions

### The *ot1* phenotype differed from those of known lower-tiller wheat mutants

The mutants *dmc* and *tin6* are characterized by mono-tillering and dwarfism from the seedling stage to the heading stage (An et al. 2019; Schoen et al. 2023). In contrast, the *ot1* mutant maintained an average of three tillers. This indicated that it retained the ability to form tillers and that the tiller inhibitory effect in this mutant therefore differed from those in other mutants. In *tin4* mutants, the tiller number is significantly decreased at the seedling stage and does not increase at all until the heading stage (Wang et al. 2022). This similar to the variation in tiller number observed in *ot1*, but plant height and spike length are not changed in *tin4*. *ftin* possesses normal tillering ability at the seedling stage but has a reduced number of effective tillers after heading (Zhang et al. 2013), which again differs from *ot1*. Previous studies have observed that reductions in tiller number result from inhibition of tiller bud differentiation or growth. To understand the mechanisms leading to reduced tiller number in *ot1*, structural variations in the *ot1* tiller buds must be examined from the very beginning of the seedling stage.

### Hormone biosynthesis and signal transduction pathways were enhanced in *ot1*

Phytohormones are some of the important factors that regulate plant growth and development. Tiller bud growth and differentiation are regulated by multiple genes and hormones, including SLs (Al-Babili and Bouwmeester 2015). Canonical SLs are primarily associated with inter-root communication in plants, whereas non-canonical SLs mainly affect tiller differentiation and growth (Ito et al. 2022). Non-canonical SLs are formed by D27, D17, and D10, then are converted to canonical SLs through a multi-step biosynthetic process involving MAX1 (Zhang et al. 2014). *ot1* tiller buds showed significant upregulation of *TaD27*, *TaD17*, *TaD10*, and *TaMAX1* at the regreening stage, demonstrating enhancement of the SL biosynthesis pathway in *ot1*. Compared with the regreening stage, *TaMAX1* was upregulated at the jointing stage. This indicated that canonical SL biosynthesis was enhanced at the jointing stage. SL signaling relies on a complex formed by D53, D14, and D3 (Shabek et al. 2018). *TaD14* and *TaD53* were significantly upregulated in *ot1* compared to the WT, indicating enhancement of the SL signaling pathway in addition to the biosynthetic pathway. *OsCKX9* functions downstream of *D53* and is involved in SL signaling. *OsCKX9* is significantly upregulated in response to increased endogenous SL content or to treatment with the SL analogue GR24 (Duan et al. 2019). *IPA1* also functions downstream of *D53* and regulates tillering in rice (Song et al. 2017). *D53* and *TaCKX9* were significantly upregulated in *ot1* compared to WT plants, whereas *IPA1* was downregulated. These outcomes were consistent with previous findings in rice, demonstrating the enhanced function of the SL signaling pathway in *ot1* tiller buds.

ABA reportedly plays an important role in inhibiting tillering and branch growth (Abuauf et al. 2018; Liu et al. 2020). Increased endogenous ABA contents can reduce tillering in wheat, rice (Liu et al. 2020), and *Arabidopsis* (Yao and Finlayson 2015). *NCED3* is a key gene in the ABA biosynthetic pathway; compared with the WT, the *Arabidopsis* mutant *nced3* has longer axillary buds and more branches (González-Grandío et al. 2017). Here, *ABA1*, *ABA2*, *ABA3*, *ABA4*, *NCED3*, and *NCED5* were all upregulated in *ot1* at both sampling stages compared with the WT. The expression levels of *NCED3* and *NCED5* were also significantly increased at the jointing compared to the regreening stage. These results demonstrate increased expression of ABA biosynthesis genes in *ot1* in addition to gradual increases in ABA synthesis gene expression during tiller development. In wheat, ABA signal transduction is dependent on PYLs, PP2Cs, and SnRK2s (Bhatnagar et al. 2017; Kim et al. 2012; Chen et al. 2020). *PP2C*s and *SnRK2*s were here found to be significantly upregulated in *ot1*, suggesting enhancement of the ABA signaling pathway. However, there were no differences in the expression of ABA signaling genes between the two sampled stages in *ot1*.

IAA accumulation inhibits tiller bud growth. The IAA efflux genes *PIN2*, *PIN9*, and *PIN5b* were differentially expressed in *ot1* compared to the WT. PIN2 and PIN9 regulate IAA transportation out of the tiller bud and promote tiller bud growth (Chen et al. 2012; Hou et al. 2021). *PIN2* and *PIN9* were upregulated in *ot1* mutant, suggesting that these genes did not negatively affect *ot1* tiller growth. In contrast to PIN2 and PIN9, PIN5b negatively regulates of tiller number. *PIN5b* overexpression significantly decreases tiller number, whereas *pin5b* mutants show a greater tiller number (Lu et al. 2015). Notably, *TaPIN5b* was upregulated at both stages in *ot1*. Further research is required to determine whether this gene affects tiller growth in *ot1*.

### Reduced tiller number in *ot1* may not be controlled by currently known tillering genes

To identify genes controlling tiller development in *ot1*, we analyzed the expression patterns of known homeologs of tiller development genes in rice and wheat. *HAP2E* overexpression increases tiller number (Alam et al. 2015). *PGL/CAO1* promotes axillary bud growth (Yang et al. 2016). *MOC1* and *FON1* promote tiller growth (Moon et al. 2006; Shao et al. 2019). These genes were significantly upregulated in *ot1* and were more highly expressed at the jointing stage compared with the regreening stage. This indicated that the effects of these genes were gradually enhanced as tiller development progressed, but that they did not increase the tiller number.

*OsbHLH025* and *IPA1* overexpression decreases tiller number (Yamamura et al. 2015; Song et al. 2017), suggesting that repression of both genes would lead to a greater tiller number. We here found that both genes were significantly downregulated in the *ot1* mutant at both stages. The tiller number, plant height, and spike length of *ot1* plants were similar to those of *tn1* mutants (Dong et al. 2023). However, *TN1* expression was not altered in *ot1* (Fig. 7c), meaning that the gene regulating oligo-tillering in *ot1* differed from the one in *tn1* and in other known tillering mutants.

## Conclusions

We have found that tiller number was significantly decreased in *ot1* mutants compared to the WT at the seedling stage, but that thousand-grain weight was significantly increased. *ot1* had a distinct phenotype compared to other known wheat oligo-tillering mutants. RNA-seq of the tiller bud revealed upregulation of genes associated with SL and ABA biosynthesis and signaling. Tiller number inhibition in *ot1* may therefore have been related to the activity of plant hormone pathways. Known tiller growth-promoting genes were significantly upregulated, whereas genes known to inhibit tiller development were downregulated in *ot1*. Thus, *ot1* may reveal a novel locus for tiller number inhibition.

### Supplementary Information

Below is the link to the electronic supplementary material.

Supplementary file1 (DOCX 580 KB)

Supplementary file2 (XLSX 313 KB)

## Data Availability

All data generated or analyzed in this study are included in the manuscript and the supplementary information files. The RNA-seq data were deposited to the National Center for Biotechnology Information (NCBI) SRA repository under accession number PRJNA996360.
