# Supplementary figures and images for: Strigolactone and abscisic acid synthesis and signaling pathways are enhanced in the wheat oligo-tillering mutant ot1
Source: Mol Breed. 2024 Feb 2;44(2):12. doi: 10.1007/s11032-024-01450-3 (PMC10837411; doi:10.1007/s11032-024-01450-3)

**Figure S1**


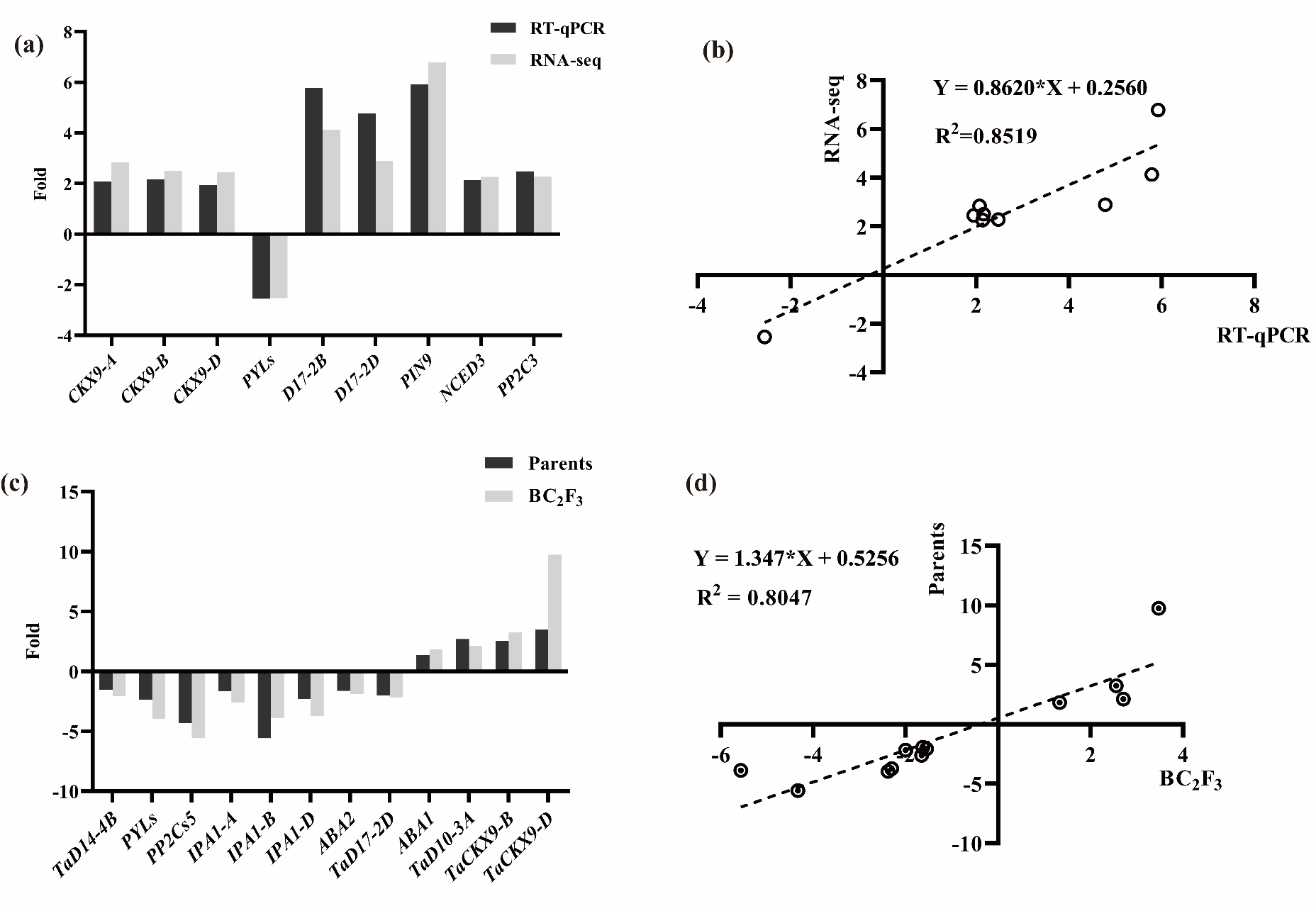


**Figure S2**


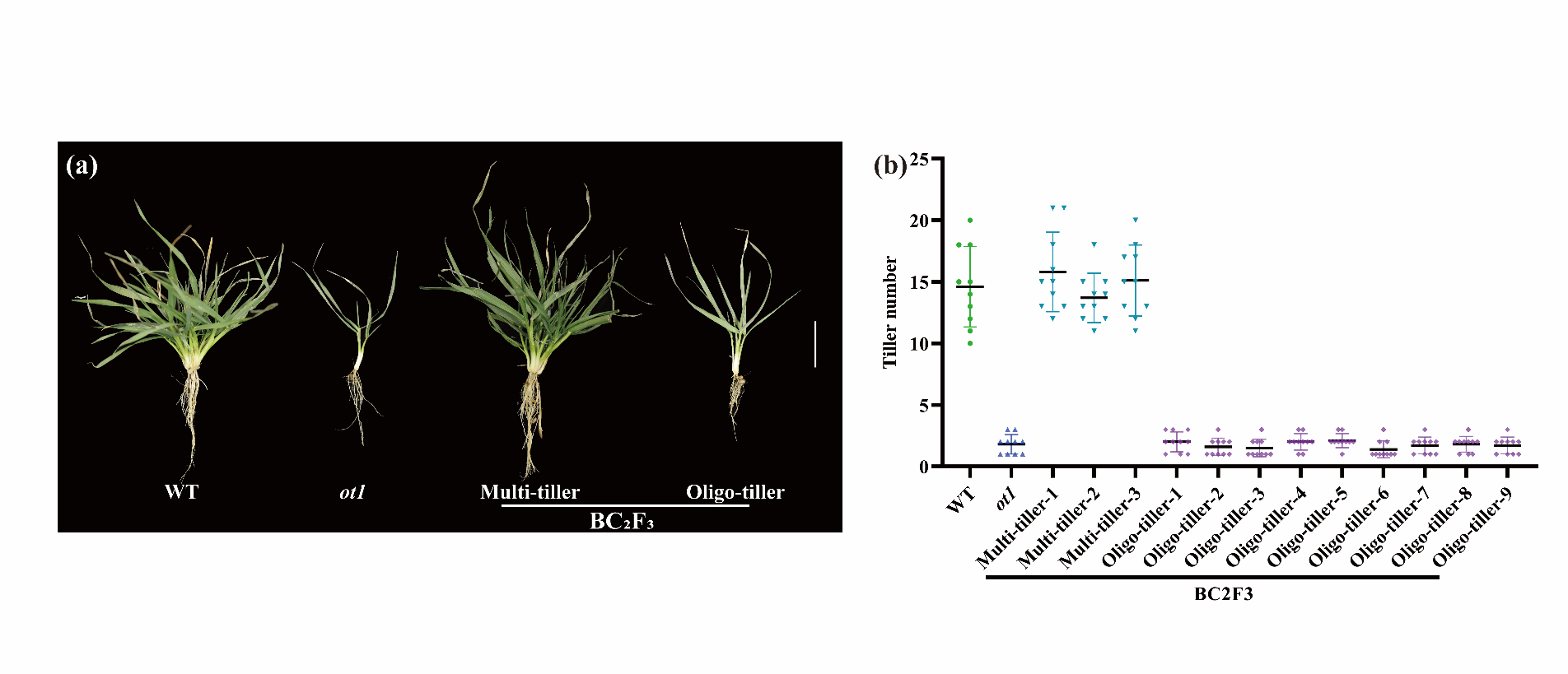

Supplement: Supplementary file 1 — Supplementary file1 (DOCX 580 KB) [file 11032_2024_1450_MOESM1_ESM.docx]
